# Interferon-γ and Smac mimetics synergize to induce apoptosis of lung cancer cells in a TNFα-independent manner

**DOI:** 10.1186/s12935-018-0579-y

**Published:** 2018-06-14

**Authors:** Qin Hao, Hua Tang

**Affiliations:** Department of Cellular and Molecular Biology, The University of Texas Health Science Center at Tyler, 11937 US Highway 271, Tyler, TX 75708 USA

**Keywords:** IFNγ, Smac mimetic, Lung cancer, Apoptosis, Inhibitor of apoptosis

## Abstract

**Background:**

The prognosis of lung cancer is very poor and hence new therapeutic strategies are urgently desired. In this study, we searched for efficacious Smac mimetic-based combination therapies with biomarkers to predict responses for non-small cell lung cancer (NSCLC).

**Methods:**

NSCLC cell lines and normal human alveolar epithelial cells were treated with Smac mimetics plus IFNγ or other agonists and cell viabilities were assessed by MTS assay, cell counting, flow cytometry and cell colony assay. Western blot analysis was performed to assess the cleavage (activation) of caspases and expression of signaling molecules. Caspase activity was determined to verify caspase activation. The pathways involved in NSCLC cell death were investigated using specific inhibitors.

**Results:**

We found that IFNγ could cooperate with various Smac mimetics to trigger a profound apoptosis in a number of NSCLC cell lines that are competent for IFNγ signaling (i.e. expressing IFNγ receptor-1 and STAT1) but have low expression levels of inhibitor of apoptosis proteins survivin and livin without harming normal human lung epithelial cells. IFNγ co-treatment with a novel class dimeric Smac mimetic AZD5582 eradicated NSCLC cell colony formation. Unlike IFNγ, IFNα, IFNλ, TNFα, or TRAIL alone or plus AZD5582 had minor effects on NSCLC cell viability. IFNγ/AZD5582-induced cell death in NSCLC cells was independent of TNFα autocrine but relied on apoptosis mediated by JAK kinase, caspase 8 and RIPK1 pathways.

**Conclusion:**

Our results indicate that IFNγ and Smac mimetics can synergize to induce apoptosis of NSCLC cells and suggest that IFNγ and Smac mimetic regimen may be a novel and efficacious apoptosis targeted therapy with biomarkers to predict responses for NSCLC cells.

## Background

Lung cancer is the leading cause of cancer mortality worldwide and contributes to about 30% of all cancer deaths [1]. Lung cancer can be divided into non-small cell lung cancer (NSCLC) which comprises 80–85% of total lung cancer cases, and the small cell lung cancer (SCLC) for the remaining 15–20% cases [2]. Attempts have been made to develop effective therapeutic strategies to combat lung cancer, such as the identification of mutations in epidermal growth factor receptor (EGFR), K-Ras, and p53, and the EGFR-targeted therapy; however over 5 years prognosis is about 16% in NSCLC and ever lower for SCLC [3]. Lung cancer therefore represents a significant clinical challenge, clearly new therapeutic strategies are urgently needed.

Resistance to apoptosis is a hallmark of many solid tumors, including lung cancer. Thus, targeting apoptotic pathway represents a promising approach that aims to selectively kill cancer cells while sparing normal ones. Apoptosis, a programmed cell death in which dying cells are phagocytized prior to membrane damage, is a physiological and non-inflammatory process that differs from necroptosis [4]. Apoptosis is tightly regulated by inhibitor of apoptosis (IAP) proteins, a family of anti-apoptotic proteins that are highly expressed in various human cancers [5]. The function of IAP proteins is antagonized by second mitochondria-derived activator of caspases (Smac) that is released from mitochondria into the cytosol during apoptosis [6]. This led to the development of a series of small molecule Smac mimetics that neutralize X-linked IAP (XIAP), cellular IAP1 (cIAP1) and cIAP2 to enhance cancer cell susceptibility to apoptosis [5]. It has been shown that Smac mimetic sensitizes NSCLC cells to multiple chemotherapy agents in an IAP-dependent but tumor necrosis factor-α (TNFα)-independent manner [7]. This finding is further confirmed by recent studies using different Smac mimetics in NSCLC [8, 9]. IAP-targeted therapies in lung cancer by using Smac mimetics, antisense oligonucleotides and gene expression inhibitors have been evaluated in clinical phase I/II trials [10–12]. Other apoptosis targeted therapies in lung cancer, including therapies targeting TNF-related apoptosis-inducing ligand (TRAIL) receptor and Bcl-2, have also gone through evaluation in preclinical and clinical phase I/II trials [10]. Although the efficacy of monotherapy appears to be limited, the apoptotic drugs seem to be promising especially in combination with other traditional therapies [10]. However, these studies lack of identification of lung cancer patient subgroups who will most likely benefit from specific apoptosis targeted therapies. Biomarkers that can predict responses to apoptosis targeted agents are important and remain to be identified.

In this study, we searched for efficacious Smac mimetic-based combination therapies with biomarkers to predict responses for lung cancer. We show that a number of human NSCLC cell lines that are competent for interferon-γ (IFNγ) signaling (i.e. expressing IFNγ receptor-1 and STAT1) but have low expression levels of IAP proteins survivin and livin, can be readily killed through apoptosis by IFNγ and Smac mimetic co-treatment without harming normal human lung epithelial cells.

## Methods

### Smac mimetics and reagents

The Smac mimetic AZD5582 was obtained from Chemietek (Indianapolis, IN, USA) and Smac mimetics SM164, BV6, and Birinapant (TL32711) were from APExBIO (Houston, TX, USA). Recombinant human IFNα was from PBL Assay Science (Piscataway, NJ, USA) and IFNγ, IFNλ, TNFα, and Annexin V-FITC were from eBioscience (San Diego, CA, USA). Recombinant human TRAIL was from ProSpec TechnoGene (East Brunswick NJ, USA). Polyinosinic–polycytidylic acid (poly(I:C)) was from InvivoGen (San Diego, CA, USA). Necrostatin-1, necrosulfonamide, GSK872, Bay11-7082, JAK kinase inhibitor I, AG-1478, and cisplatin were from EMD Millipore (Billerica, MA, USA). The general caspase peptide inhibitor Z-VAD-FMK and the caspase-8 peptide inhibitor Z-IETD-FMK were from R & D Systems (Minneapolis, MN, USA). Human TNFα neutralizing antibody (#7321) was from Cell Signaling Technology (Beverly, MA, USA). Caspase-3 and -8 colorimetric assay kits were from BioVision (Milpitas, CA, USA). All other chemicals were obtained from Sigma-Aldrich (St. Louis, MO, USA).

### Cell culture and cell viability assay

Human NSCLC cell lines including NCI-H1975, NCI-H1437, NCI-H441, HCC827, A549, and Calu-3 were obtained from American Type Culture Collection (Manassas, VA, USA) and cultured in RPMI-1640 medium supplemented with 10% fetal calf serum. Primary normal human alveolar epithelial cells were from Cell Biologics (Chicago, IL, USA), cultured in epithelial cell growth medium, and used for experiments within four passages. For cell viability assay, cells were seeded into 48-well plates, grown to subconfluence, then incubated with various agonists in the presence or absence of Smac mimetics, caspase or kinases inhibitors for indicated time periods. Cell viability was assessed by MTS assay using CellTiter AQ_ueous_ one solution reagent according to the manufacturer’s instructions (Promega, Madison, WI, USA) and cell counting with trypan blue by a TC20 automated cell counter. Cell survival rate was calculated by comparison to DMSO-treated control cells and are presented as mean ± SE (n = 3).

### Cell colony assay

Cells were seeded into 12-well plates at 500 cells/well overnight and then incubated with different agonists in the presence of control DMSO or Smac mimetics for 4 weeks. Media were changed every 5 days. Cell colonies were fixed and stained with 0.05% crystal violet and the stain was eluted and quantified at 540 nm.

### Western blot analysis

Western blot analysis was performed essentially as we described previously [13]. The membranes were probed with the following primary antibodies against: phospho-receptor-interacting protein kinase-1 (RIPK1) (Ser166) (#65746), cleaved caspase-3 (#9664), -7 (#8438), -8 (#9496), -9 (#7237), cleaved poly (ADP-ribose) polymerase (PARP) (#9541), cIAP-1 (#7065), cIAP-2 (#3130), XIAP (#2045), survivin (#2808), livin (#5471), STAT1 (#9712), phospho-STAT1 (#7649), STAT3 (#9139), JAK1 (#3332), JAK2 (#3229) (Cell Signaling Technology, Beverly, MA, USA), IFNγ receptor 1 (#AF673) (R & D Systems, Minneapolis, MN, USA), EGFR (#sc-03) (Santa Cruz Biotechnology, Santa Cruz, CA, USA), vinculin (#V9131) and actin (#A4700) (Sigma. St. Louis, MO, USA). The membranes were then incubated with horseradish peroxidase-conjugated secondary antibodies (Cell Signaling Technology) and detected with Bio-Rad Clarity Western ECL substrate (Hercules, CA, USA).

### Annexin V apoptosis assay

Cells were seeded into 6-well plates, grown to subconfluence, then incubated with AZD5582 and IFNγ for 48 h. Floating dead cells were removed and the adherent cells were rinsed and harvested with a diluted trypsin solution. Cells were then stained with FITC Annexin V to identify cells undergoing apoptosis by flow cytometry.

### Caspase-3 and -8 activity assay

Cell lysates (100 μg) were incubated with 200 μM of caspase-3 substrate DEVD-chromophore p-nitroaniline (pNA) or caspase-8 substrate IETD-pNA in 2× reaction buffer, and pNA light emission was detected at 405 nm according to the manufacturer’s protocol (BioVision) and presented as OD_405 nm_ over 500 μg protein.

### Statistical analysis

Data are expressed as mean ± SE. Statistical analyses were performed using Microsoft Excel and GraphPad Prism (GraphPad Software, La Jolla, CA, USA). Data were analyzed by Student’s *t* test. p < 0.05 is considered statistically significant.

## Results

### IFNγ cooperates with Smac mimetics to trigger a TNFα-independent apoptosis in the H1975 NSCLC cell line

**Fig. 1:**
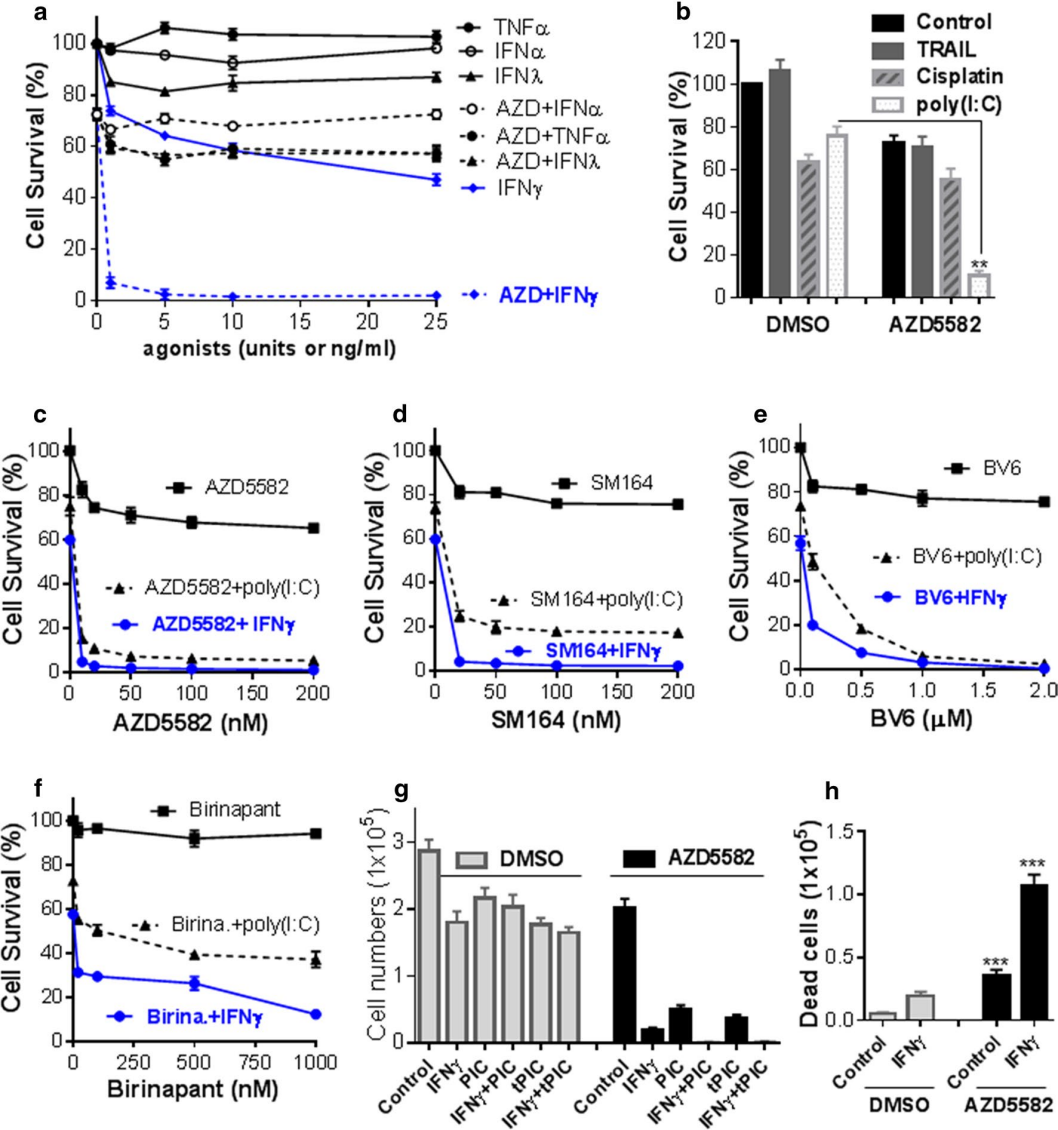
IFNγ and Smac mimetics synergistically induce cell death in the H1975 NSCLC cell line. **a**, **b** H1975 NSCLC cells were incubated with 1–25 units of human IFNα or 1–25 ng/ml of IFNγ, IFNλ or TNFα, TRAIL (500 ng/ml), cisplatin (25 µM) or poly(I:C) (500 ng/ml) in the presence or absence of 20 nM AZD5582 for 48 h. **c**–**f** H1975 cells were incubated with 10 ng/ml IFNγ or 250 ng/ml poly(I:C) plus different doses of AZD5582 (**c**), SM164 (**d**), BV6 (**e**) or Birinapant (**f**) for 48 h. Cell viabilities (**a**–**f**) were assessed by MTS assay and cell survival rates were calculated by comparison to DMSO-treated control cells and are presented as mean ± SE (n = 3). **g** H1975 cells were treated with IFNγ (10 ng/ml), poly(I:C) (PIC, 500 ng/ml), IFNγ plus poly(I:C), or transfected with poly(I:C) (tPIC, 100 ng/ml) alone or plus IFNγ in the presence or absence of 20 nM AZD5582 for 48 h. Viable cells were counted with trypan blue by a TC20 automated cell counter (n = 3). **h** H1975 cells were treated with IFNγ (5 ng/ml) in the presence of DMSO or AZD5582 (20 nM) for 48 h, and the detached cells were collected and counted with trypan blue by a TC20 automated cell counter (n = 3). **p < 0.01; ***p < 0.001 versus DMSO. Results represent the findings of three independent experiments

**Fig. 2:**
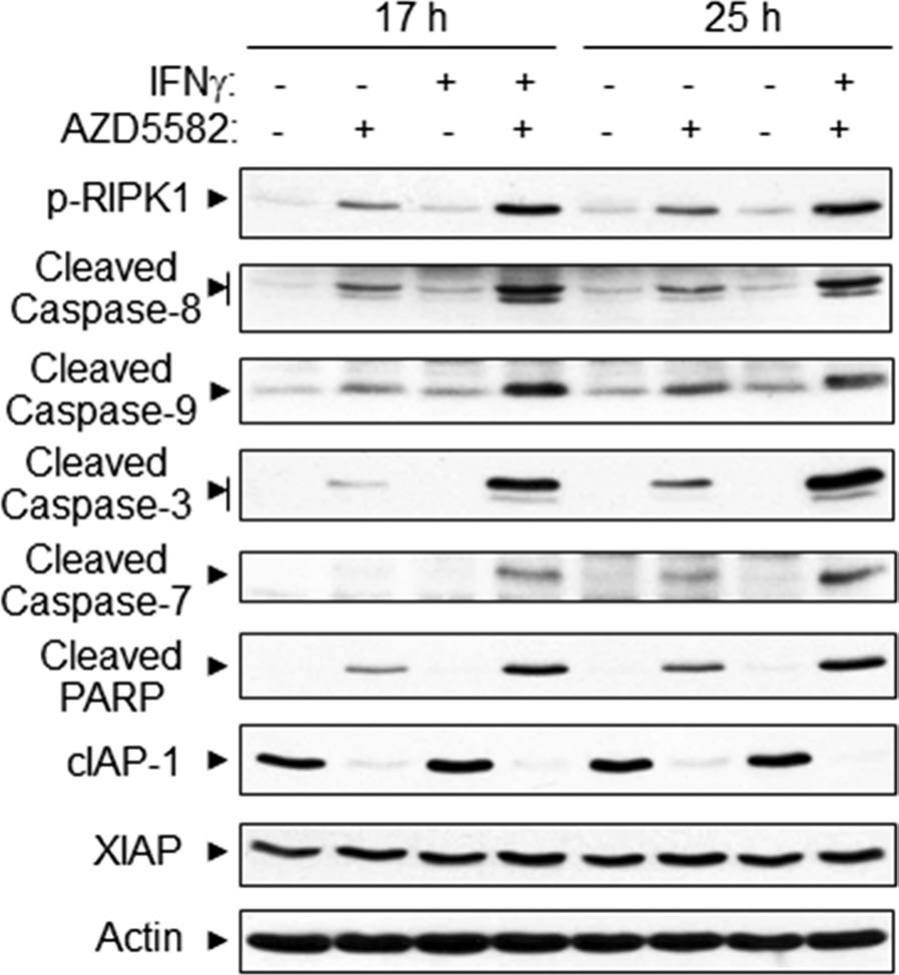
IFNγ and Smac mimetic AZD5582 synergistically induce caspase activation in H1975 NSCLC cell line. H1975 cells were treated with IFNγ (10 ng/ml) and AZD5582 (20 nM) for 17 or 25 h, and cell lysates at equal amounts were subjected to Western blotting with indicated antibodies. Results represent Western blots of three independent experiments

**Fig. 3:**
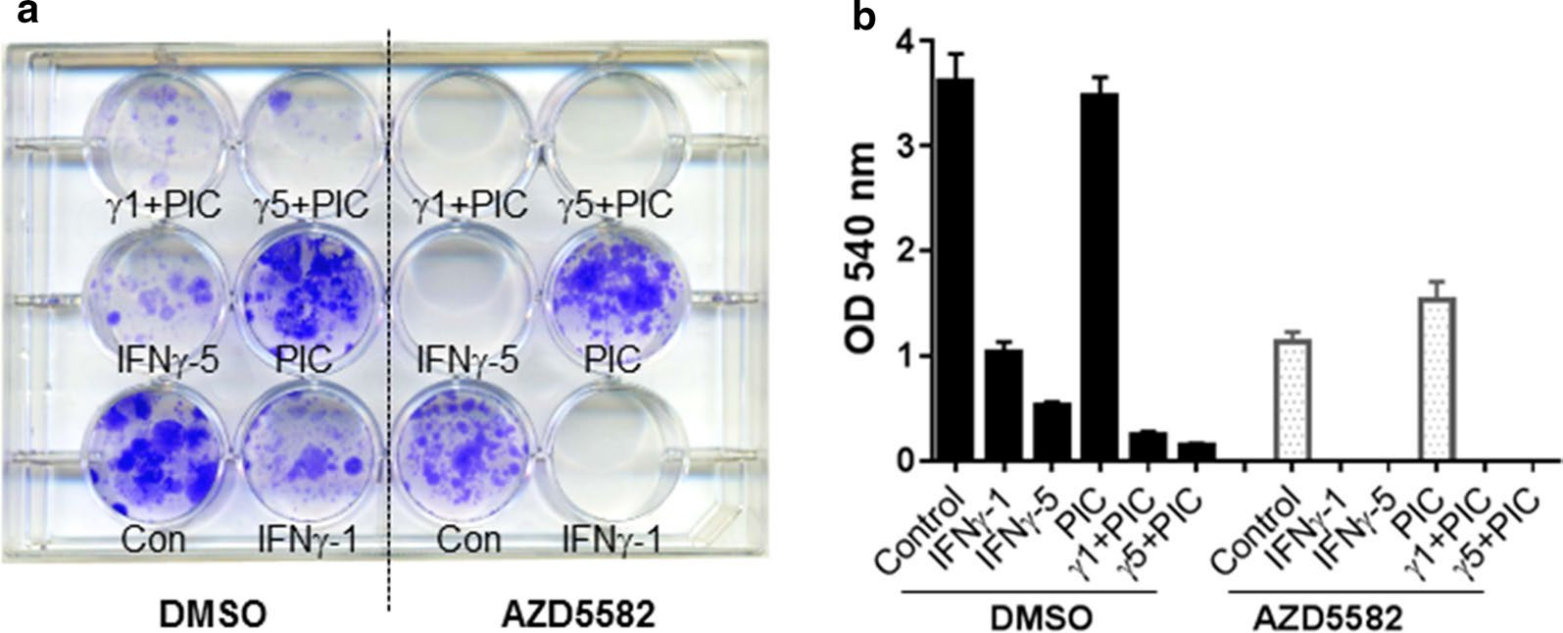
No H1975 cell colony survives by co-treatment of IFNγ with Smac mimetic AZD5582. H1975 cells were incubated with 1 or 5 ng/ml IFNγ (IFNγ-1 or IFNγ-5), poly(I:C) (PIC, 250 ng/ml), or poly(I:C) (250 ng/ml) with 1 or 5 ng/ml IFNγ (γ1 + PIC or γ5 + PIC) in the presence of control DMSO or AZD5582 (20 nM) for 4 weeks. Cell colonies were stained with crystal violet shown in plate (**a**) and crystal violet stain was eluted and quantified at OD540 nm (**b**). Results represent three independent experiments

As shown in Fig. 1a, we treated H1975 human NSCLC cell line harboring EGFR T790 M and L858R mutations with AZD5582 [14], a novel class of dimeric Smac mimetics, plus various agonists for 48 h and the cell viability was assessed. We found that AZD5582 alone at 20 nM slightly inhibited cell viability, but it could cooperate with IFNγ to profoundly induce cell death even with IFNγ at 1 ng/ml. In contrast, AZD5582 barely induced such synergetic effects with TNFα, IFNα, or IFNλ. As expected, IFNγ alone reduced cell viability dose-dependently, which might be due to the direct inhibition of cell proliferation and induction of apoptosis [15]. Interestingly, AZD5582 also cooperated with poly(I:C), a synthetic analog of viral double-stranded RNA (dsRNA) to induce cell death, whereas AZD5582 had a minor effect on cell death by cisplatin or TRAIL (Fig. 1b). We further showed that IFNγ or poly(I:C) not only cooperated with AZD5582 but also with other Smac mimetics including SM164 [16], BV6 [17] and Birinapant [18] to markedly induce cell death, and that IFNγ appeared to have a stronger effect compared with poly(I:C) (Fig. 1c–f). Birinapant is a monovalent Smac mimetic and its synergetic effect was weaker than other three bivalent Smac mimetics. Moreover, cell counting with trypan blue confirmed the synergetic effects on cell death induced by AZD5582 plus IFNγ or poly(I:C) (Fig. 1g, h). Additionally, AZD5582 plus IFNγ and poly(I:C) appeared to have a stronger effect on cell death than AZD5582 plus IFNγ or AZD5582 plus poly(I:C) (Fig. 1g). To assess contribution of apoptosis to the cell death, we performed Western blots analysis and found that AZD5582 alone down-regulated cIAP-1 but not XIAP, activated RIPK1 [19] that is an important upstream regulator of caspase-8, and triggered the cleavage (activation) of extrinsic (caspase-8) and intrinsic (caspase-9) apoptosis pathways, causing the cleavage (activation) of caspase-3 and caspase-7, the primary executioners of apoptosis, and of DNA repair enzyme PARP, one of the main cleavage targets of caspase-3 (Fig. 2). Importantly, the apoptosis-inducing effect of AZD5582 was markedly enhanced by co-treatment with IFNγ (Fig. 2). These findings suggest that IFNγ and Smac mimetics synergistically kill H1975 NSCLC cells likely through apoptosis. To assess the long term effect on cell growth, we performed colony formation assay and found that no cell colony could survive by co-treatment of AZD5582 with IFNγ at 1 or 5 ng/ml (Fig. 3). In contrast, a great number of colonies formed in medium containing AZD5582 alone or AZD5582 plus poly(I:C). IFNγ alone or IFNγ plus poly(I:C) markedly inhibited clonogenic growth, but could not eradicate the colony formation (Fig. 3).

**Fig. 4:**
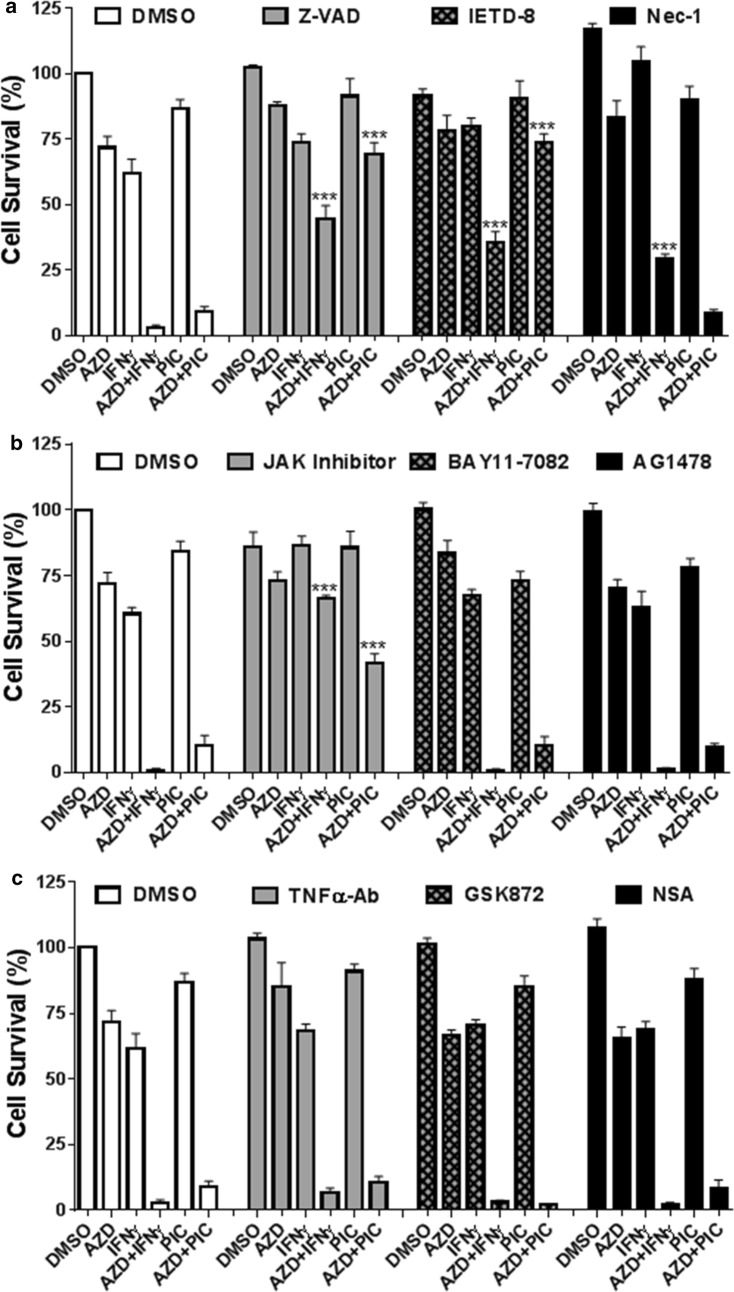
IFNγ/AZD5582-induced apoptosis is mediated by JAK kinase but not TNFα production in H1975 NSCLC cell line. H1975 cells were incubated with DMSO, AZD5582 (20 nM), IFNγ (10 ng/ml), poly(I:C) (PIC, 250 ng/ml), AZD5582 plus IFNγ (AZD + IFNγ), or AZD5582 plus poly(I:C) (AZD + PIC) in the presence DMSO, Z-VAD-FMK (25 µM), Z-IETD-FMK (IETD-8, 25 µM), necrostatin-1 (Nec-1, 40 µM), JAK inhibitor-1 (500 nM), BAY11-7082 (5 µM), AG1478 (250 nM), TNFα neutralizing antibody (TNFαAb, 1 µg/ml), GSK872 (5 µM), necrosulfonamide (NSA, 1 µM) for 48 h. Cell viabilities were assessed by MTS assay and cell survival rates were calculated by comparison to DMSO-treated control cells (n = 3). ***p < 0.001 versus DMSO. Results represent the findings of three independent experiments

We further examined the molecular basis of cell death by AZD5582 co-treatment with IFNγ or poly(I:C) in H1975 cells. We found that AZD5582/IFNγ-induced cell death was almost blocked by a specific JAK kinase inhibitor [20] and markedly prevented by a general caspase inhibitor Z-VAD-FMK [21], a specific caspase-8 inhibitor Z-IETD-FMK [22] and a selective RIPK1 inhibitor necrostatin-1 [23] (Fig. 4a, b). While AZD5582/poly(I:C)-induced cell death was markedly suppressed by Z-VAD-FMK and Z-IETD-FMK, partially suppressed by the specific JAK kinase inhibitor, but was not affected by necrostatin-1 (Fig. 4a, b). Moreover, cell death induced by AZD5582 plus IFNγ or poly(I:C) was essentially not affected by a NK-κB inhibitor BAY11-7082, an EGFR inhibitor AG1478, a human TNFα neutralizing antibody, a specific inhibitor (GSK872) of necroptosis initiator RIPK3 [24], or a specific inhibitor (necrosulfonamide) of necroptosis effector mixed lineage kinase domain-like protein (MLKL) [25] (Fig. 4b, c). These findings indicate that AZD5582/IFNγ-induced cell death in H1975 NSCLC cell line is mediated by JAK kinase through apoptosis pathway independent of TNFα autocrine and necrotic cell death (necroptosis).

### IFNγ and Smac mimetics synergistically induce apoptosis in IFNγ signaling competent NSCLC cell lines

**Fig. 5:**
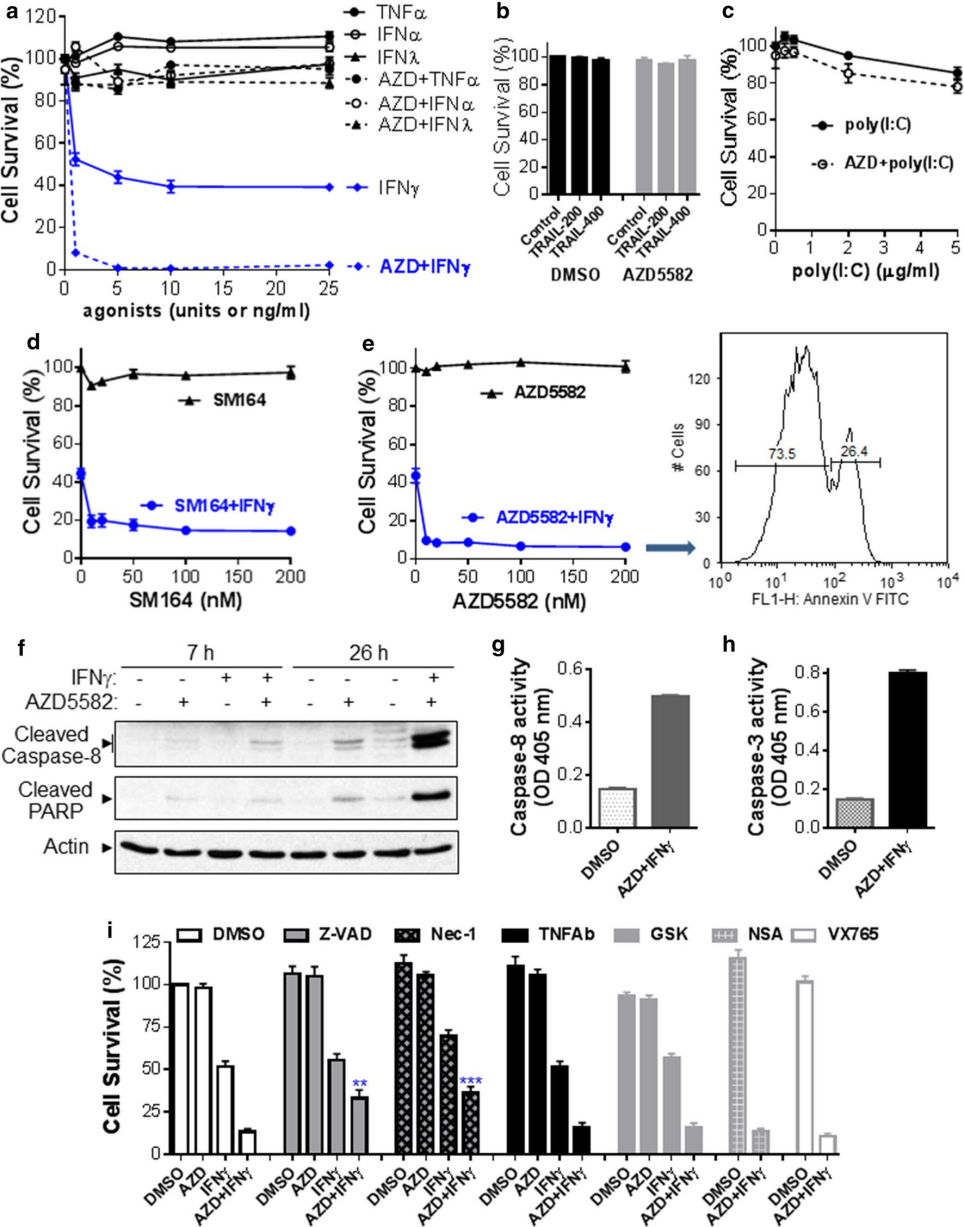
IFNγ and Smac mimetics synergistically induce apoptosis in HCC827 NSCLC cell line. **a**, **b** HCC827 NSCLC cells were incubated with 1–25 units of human IFNα or 1–25 ng/ml of IFNγ, IFNλ or TNFα (**a**), or with TRAIL (200 or 400 ng/ml) (**b**) in the presence or absence of 20 nM AZD5582 for 48 h. **c** HCC827 cells were incubated with various doses of poly(I:C) in the presence or absence of 20 nM AZD5582 for 48 h. **d**, **e** HCC827 cells were incubated with 5 ng/ml IFNγ plus different doses of SM164 or AZD5582 for 48 h. The remaining adherent cells after 48 h treatment with AZD5582 (20 nM) plus IFNγ (5 ng/ml) were subjected to Annexin V apoptosis assay (**e**). **f** HCC827 cells were treated with IFNγ (5 ng/ml) and AZD5582 (20 nM) for 7 or 26 h, and cell lysates at equal amounts were subjected to Western blotting with indicated antibodies. **g**, **h** HCC827 cells were treated with IFNγ (5 ng/ml) and AZD5582 (20 nM) for 24 h and caspase-8 and -3 activities were determined. **i** HCC827 cells were incubated with DMSO, AZD5582 (20 nM), IFNγ (5 ng/ml) or AZD5582 plus IFNγ (AZD + IFNγ) in the presence DMSO, Z-VAD-FMK (25 µM), necrostatin-1 (Nec-1, 25 µM), TNFα neutralizing antibody (TNFαAb, 1 µg/ml), GSK872 (GSK, 5 µM), necrosulfonamide (NSA, 1 µM), or VX-765 (10 μM) for 48 h. Cell viabilities (**a**–**e**, **i**) were assessed by MTS assay and cell survival rates were calculated by comparison to DMSO-treated control cells (n = 3). **p < 0.01; ***p < 0.001 versus DMSO. Results represent three independent experiments

Similar to the H1975 cell line, we found that Smac mimetics AZD5582 or SM164 cooperated with IFNγ, but not with TNFα, IFNα, IFNλ, TRAIL or poly(I:C) to induce cell death in HCC827 NSCLC cell line overexpressing EGFR and harboring L858R mutation (Fig. 5a–e). It should be noted that Smac mimetics AZD5582 and SM164 alone or AZD5582 plus poly(I:C) barely had effects on cell viability in HCC827 cells (Fig. 5c–e), which is different from the observation in H1975 cells. Majority of HCC827 cells were killed by co-treatment of AZD5582 (20 nM) plus IFNγ (5 ng/ml) for 48 h, resulting in a large amount of detached dead cells. The remaining adherent cells were subjected to Annexin V apoptosis assay; and we found that 26.4% of cell populations were detected with a higher Annexin V binding, indicating cells undergoing apoptosis (Fig. 5e, right panel). We further found that AZD5582 and IFNγ synergistically induced a time-dependent cleavage (activation) of caspase-8 and PARP in HCC827 cells (Fig. 5f). We also measured caspase-3 and -8 activities using specific peptide substrates and found that both caspases were markedly activated by AZD5582 and IFNγ co-treatment in HCC827 cells (Fig. 5g, h). Moreover, we found that AZD5582/IFNγ-induced cell death in HCC827 cells was markedly suppressed by a general caspase inhibitor Z-VAD-FMK [21] and a selective RIPK1 inhibitor necrostatin-1 [23], indicating the involvement of apoptosis (Fig. 5i). In contrast, the AZD5582/IFNγ-induced cell death was essentially not affected by a human TNFα neutralizing antibody, the RIPK3 inhibitor GSK872 [24], MLKL inhibitor necrosulfonamide [25], or a selective caspase-1 inhibitor VX-765 [26].

**Fig. 6:**
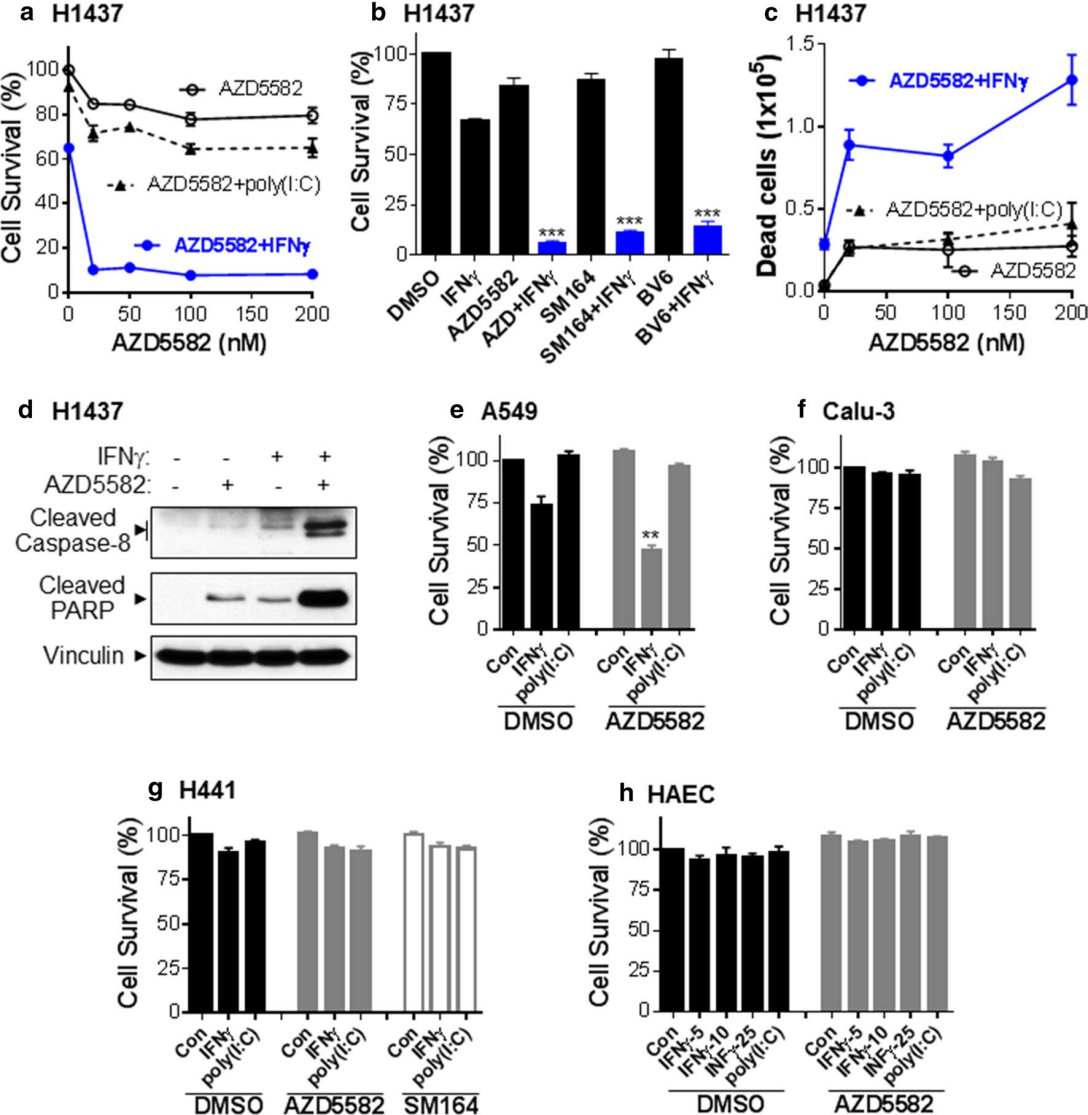
Effects of IFNγ and Smac mimetics on cell death in other NSCLC cells and HAECs. **a**, **b** H1437 cells were incubated with 10 ng/ml IFNγ or 250 ng/ml poly(I:C) plus different doses of AZD5582 (**a**) or with 10 ng/ml IFNγ alone or plus 20 nM AZD5582, 20 nM SM164, or 250 nM BV6 (**b**) for 60 h, and cell viabilities were assessed. **c** H1437 cells were treated with IFNγ (10 ng/ml) or poly(I:C) (250 ng/ml) plus different doses of AZD5582 for 60 h, and the detached cells were collected and counted with trypan blue by a TC20 automated cell counter (n = 3). **d** H1437 cells were treated with IFNγ (10 ng/ml) and AZD5582 (20 nM) for 27 h, and cell lysates at equal amounts were subjected to Western blotting with indicated antibodies. **e**–**h** A549, Calu-3 and H441 NSCLC cell lines or HAECs were treated with IFNγ (25 ng/ml) or poly(I:C) (250 ng/ml) or with the indicated amounts of IFNγ (**h**) in the presence of DMSO or 20 nM of AZD5582 or SM164 for 72 h. Cell viabilities (**a**–**h**) were assessed by MTS assay and cell survival rates were calculated by comparison to DMSO-treated control cells (n = 3). **p < 0.01, ***p < 0.001 versus DMSO. Results represent three independent experiments

The synergetic effects of IFNγ with Smac mimetics AZD5582, SM164 or BV6 on cell death was also observed in NSCLC cell line H1437 harboring p53 mutation; however Smac mimetics alone or AZD5582 plus poly(I:C) had only a minor effect on the cell viability (Fig. 6a–c). Western blots analysis revealed that AZD5582 and IFNγ synergistically and markedly induced the cleavage (activation) of caspase-8 and PARP in H1437 cells (Fig. 6d). In A549 cells, AZD5582 alone had a minor effect on cell viability; however it could cooperate with IFNγ to induce cell death even though the magnitude was much lower compared with H1975, HCC827 and H1437 cell lines (Fig. 6e). On the contrary, Smac mimetics alone or plus IFNγ or poly(I:C) essentially did not trigger cell death in NSCLC cell lines Calu-3 and H441 and in primary normal human alveolar epithelial cells (HAECs) (Fig. 6f–h).

**Fig. 7:**
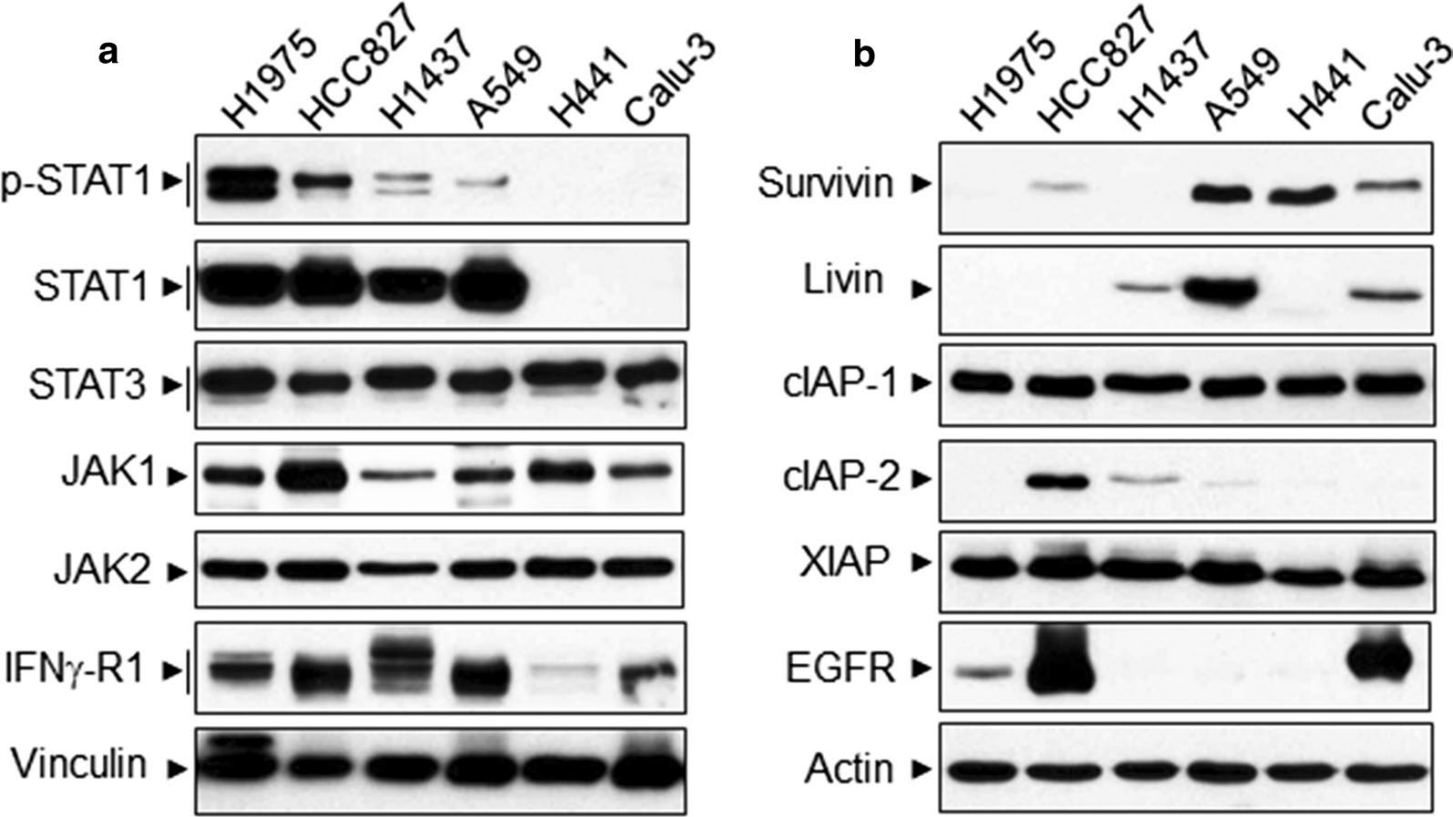
Expression levels of IFNγ signal components and IAP proteins in NSCLC cell lines. Cell lysates at equal amounts from NSCLC cell lines were subjected to Western blotting with indicated antibodies. Results represent Western blots of three independent experiments

Western blot analysis shows that NSCLC cell lines H441 and Calu-3 which were resistant to IFNγ/Smac mimetic co-treatment did not express STAT1 [27], a central molecular for IFNγ signaling (Fig. 7a, second panel). A549 cells that weakly responded to IFNγ/Smac mimetic co-treatment expressed STAT1 but had less phosphorylation of STAT1 on Tyr-701 compared with H1975, HCC827 and H1437 cell lines (Fig. 7a, first panel). Moreover, among the examined IAP proteins, survivin and livin were highly expressed in A549 cells (Fig. 7b, first and second panels). Survivin was also highly expressed in H441 and Calu-3 cells. All the examined NSCLC cell lines expressed IFNγ receptor-1, JAK1/2, STAT3 and comparable levels of cIAP-1 and XIAP (Fig. 7). In addition, primary HAECs expressed IFNγ-R1, STAT1 and JAK1/2 (data not shown). Taken together, our findings indicate that Smac mimetics and IFNγ synergistically induce apoptosis only in IFNγ signaling competent NSCLC cell lines. It also suggests that high expression levels of survivin and livin in A549 cells may render the cell not very responsive to IFNγ/Smac mimetic regimen.

## Discussion

Lung cancer represents a significant clinical challenge; hence new therapeutic strategies are urgently needed. In the present study, we report a novel finding that IFNγ cooperates with Smac mimetics to trigger a profound apoptosis in a number of human NSCLC cell lines that are competent for IFNγ signaling (i.e. expressing IFNγ receptor-1 and STAT1) but have low expression levels of IAP proteins survivin and livin without harming normal lung epithelial cells. We further show that IFNγ/AZD5582-induced cell death in NSCLC cell lines is mediated by JAK kinase through apoptosis but not necroptosis pathway independent of TNFα autocrine. Thus, IFNγ and Smac mimetic regimen may be a novel and efficacious apoptosis targeted therapy with biomarkers to predict responses for NSCLC cells.

Smac mimetics as mono- or combination therapies are currently undergoing clinical evaluation in many trials against a variety of human cancers [11, 12]. Smac mimetics when tested as single agents can induce apoptosis in cancer cells via a TNFα autocrine mechanism [28, 29], but only a small subset of cancer cells response to Smac mimetics and there is no biomarker to predict the response. AZD5582 is a novel class of dimeric Smac mimetics and its antiproliferative effect is only observed in 14 out of 200 examined cancer cell lines, consistent with other published IAP inhibitors [14]. Similarly, we found that Smac mimetics such as AZD5582, SM164 or BV6 alone only slightly (20–30%) inhibited cell viability in H1975 and H1437 cells but essentially had no effect in other 4 NSCLC cell lines. Although the efficacy of monotherapy appears to be limited, Smac mimetic-based combination anticancer therapies seem to be promising [11, 12]. It has been shown that Smac mimetic sensitizes NSCLC cells to multiple chemotherapy agents in a TNFα-independent manner [7], which is further confirmed by recent studies using different Smac mimetics in NSCLC [8, 9]. However, these studies lack of identification of subgroups of lung cancer patients who will most likely benefit from IAP-targeted therapies. We found that Smac mimetics such as AZD5582, SM164 and BV6 could cooperate with IFNγ to profoundly (> 90%) induce cell death in H1975, HCC827 and H1437 NSCLC cell lines that expressed STAT1 [27], a central molecular for IFNγ signaling. A549 cells that weakly responded to IFNγ/Smac mimetic co-treatment also expressed STAT1 but had less STAT1 phosphorylation on Tyr-701 compared with H1975, HCC827 and H1437 cell lines. In contrast, NSCLC cell lines H441 and Calu-3 that did not express STAT1 were resistant to IFNγ/Smac mimetic co-treatment. All the 6 examined NSCLC cell lines expressed IFNγ receptor-1, JAK1/2, STAT3 and comparable levels of cIAP-1 and XIAP. Thus our findings indicate that Smac mimetics and IFNγ synergistically induce apoptosis only in IFNγ signaling competent (i.e. expressing IFNγ receptor-1 and STAT1) NSCLC cell lines. It has been shown that STAT1 is one of five good genes closely associated with relapse-free and overall survival among NSCLC patients [30]. STAT1 may be a potential biomarker that can predict response to IFNγ/Smac mimetic therapy for NSCLC. Moreover, among the examined IAP proteins, we found that survivin and livin were highly expressed in A549 cells. Survivin and livin are recognized targets for cancer therapy, including lung cancer; and silencing survivin or livin increases apoptosis and sensitizes NSCLC cells to chemotherapy [31–33]. This suggests that the high expression levels of survivin and livin in A549 cells may render the cell not very responsive to IFNγ/Smac mimetic regimen and that STAT1 together with survivin and livin may be valuable biomarkers that can predict the optimal responses to IFNγ/Smac mimetic therapy for NSCLC. Importantly, we found that normal human alveolar epithelial cells were resistant to IFNγ/Smac mimetic co-treatment even though IFNγ signaling components were expressed in the cells. Hence IFNγ/Smac mimetic therapy preferentially targets IFNγ signaling competent NSCLC cells but not normal human lung epithelial cells.

Unlike type II IFNγ, IFNα, IFNλ, TNFα, or TRAIL alone or in combination with Smac mimetic AZD5582 had very minor effects on cell viability in the examined NSCLC cell lines. Interestingly, we found that dsRNA poly(I:C) and AZD5582 also synergistically induced apoptosis in H1975 cells but not in other 5 NSCLC cell lines. It has been shown that poly(I:C) also can cooperate with Smac mimetics to kill murine EMT6 breast cancer cells [34]. Poly(I:C) can be recognized by dsRNA receptors, such as endosomal Toll-like receptor 3 and cytosolic RIG1-like receptors including RIG-1, MDA5, and LGP2. Direct addition of poly(I:C) to culture medium or transfection of poly(I:C) into H1975 cells could cooperate with AZD5582 to trigger apoptosis; and the effects were potentiated by including of IFNγ. However, colony formation assay revealed that a great number of colonies still formed by the treatment of AZD5582 alone or AZD5582 plus poly(I:C). In contrast, no cell colony could survive by co-treatment of AZD5582 with IFNγ at 1 or 5 ng/ml. Collectively, these findings indicate that IFNγ/Smac mimetic therapy is much stronger and broader than AZD5582/poly(I:C) co-treatment for killing NSCLC cells. Moreover, the mechanism of AZD5582/IFNγ-induced apoptosis in H1975 cells appears to be different at some points from AZD5582/poly(I:C) co-treatment. The AZD5582/IFNγ-induced apoptosis was dependent on JAK kinase activity and mediated by caspase-8 and RIPK1; while the AZD5582/poly(I:C)-induced apoptosis was dependent on caspase-8 and regulated by JAK kinase but not RIPK1. Nevertheless, the apoptosis by AZD5582 plus IFNγ or poly(I:C) was not affected by a human TNFα neutralizing antibody, indicating a TNFα-independent mechanism. IFN-γ has antitumor activity and has been used clinically to treat a variety of malignancies, albeit with mixed results [15, 35]. As Smac mimetics have been shown to be well-tolerated in early clinical trials [11, 12], IFNγ and Smac mimetic combination therapy merits further investigation and may present a promising novel apoptosis targeted regimen for a subgroup of lung cancer cells with biomarkers to predict responses.

## Conclusions

In summary, we have shown that a number of human NSCLC cell lines that are competent for IFNγ signaling (i.e. expressing IFNγ receptor-1 and STAT1) but have low expression levels of IAP proteins survivin and livin, can be readily killed and eradicated through apoptosis by IFNγ and Smac mimetic co-treatment without harming normal human lung epithelial cells.

## Abbreviations

IFN: interferon
Smac: second mitochondria-derived activator of caspases
NSCLC: non-small cell lung cancer
IAP: inhibitor of apoptosis
EGFR: epidermal growth factor receptor
cIAP: cellular IAP
XIAP: X-linked IAP
TNF: tumor necrosis factor
TRAIL: TNF-related apoptosis-inducing ligand
poly(I:C): polyinosinic–polycytidylic acid
RIPK1: receptor-interacting protein kinase 1
PARP: poly(ADP-ribose) polymerase
